# Toward a Better Understanding of Language Learning Motivation in a Study Abroad Context: An Investigation Among Chinese English as a Foreign Language Learners

**DOI:** 10.3389/fpsyg.2022.855592

**Published:** 2022-02-28

**Authors:** Zhen Yue, Kai Zhao, Yaru Meng, Xi Qian, Lin Wu

**Affiliations:** ^1^School of Foreign Studies, Xi’an Jiaotong University, Xi’an, China; ^2^School of Economic and Finance, Xi’an Jiaotong University, Xi’an, China; ^3^Economic Research Center of Buchang Xixian, Shaanxi Institute of International Trade & Commerce, Xi’an, China; ^4^School of Foreign Studies, Wenzhou University, Wenzhou, China

**Keywords:** language learning motivation, study abroad, international posture, the ideal L2 self, Chinese EFL learners, EFL education

## Abstract

Motivation has been recognized as a vital component in successfully learning a second or foreign language. However, research on language learners’ motivation in a study abroad context requires more attention in an era in which international mobility is becoming a new normal. This study investigated 217 Chinese overseas university students’ L2 motivation during their one-year postgraduate study in the United Kingdom. by examining a range of motivational variables in relation to their motivated English language learning behaviors. Integrating results from both questionnaires and interviews from nine participating students, the study revealed that international posture showed the strongest positive power, followed by the ideal L2 self, in explaining the learners’ willingness to communicate, frequency of communication, and intended learning effort. Additionally, instrumentality and parental encouragement exerted prominent promotional influence in shaping their intended learning effort. However, the ought-to L2 self-displayed a significant negative impact on their L2 learning in this study abroad context, and the role of attitudes toward L2 speakers/community and culture was not evident in this case. The findings shed light on a more comprehensive understanding of L2 motivation in a study abroad context, and offer insightful implications for English as a Foreign Language education in cultivating language learners’ motivation to prepare for study abroad.

## Introduction

Motivation is commonly considered as a vital antecedent for accomplishing any complicated activity (Brown, 2000). It is conceived as the sum of incentives that positively empower the option of a specific behavior or purpose (Dörnyei, 2001; Jarvis, 2005). The wide recognition of motivation as a significant factor influencing the success or failure in second or foreign language (L2 or FL) learning has encouraged considerable researchers’ interest in exploring L2 motivation over the last three decades (Csizér, 2017). A large number of studies have investigated differences in motivation to learn an L2 across diverse foreign language contexts, including distinctions in L2 motivation between learners in domestic and study abroad learning contexts (Fryer and Roger, 2018). Being motivated (or not) can make all the difference in how successfully individuals learn an L2 and benefit from overseas studying experiences. However, the majority of the existing relevant studies tend to use the study abroad context as a categorical label opposed to at-home (Taguchi, 2018), by measuring either isolated individual or contextual variables in relation to L2 motivation at the expense of the other meaningful influential variables and comparing a group of learners in a study abroad context with their counterparts in a domestic setting (Farid and Lamb, 2020). These studies showed limited concern with what actually goes on with learners’ L2 motivation in a study abroad context, and therefore have not been able to allow a comprehensive understanding of the complex L2 motivation among overseas L2 learners who retain particular historical and individual contexts but are located in a milieu with increasing international mobility.

Chinese English as a Foreign Language (EFL) learners constitute the largest group in the global flow of tertiary-level students, especially in the trend of studying in a destination country where English is one of the official languages with the presence of English as the *lingua franca* (Isabelli-García et al., 2018). There is a body of studies on motivation of Chinese learners of foreign languages in domestic formal institutions (Boo et al., 2015). However, research on Chinese overseas students’ L2 motivation experiences in a study abroad context, where English is nearly ubiquitous in daily life and is determinant for both their living quality and academic success (Yu et al., 2018; Pawlak et al., 2020), requires both EFL teachers and educational policymakers’ increased attention. It should become one of primary goals in EFL education to cultivate language learners’ L2 motivation and profit from their study abroad experiences that are becoming more and more easily accessible to Chinese as well as other similar sociocultural university students.

By focusing on 217 Chinese overseas university students’ English language learning outside the instructional settings in the local community in the United Kingdom, the current study examined the complex impacts of various motivational variables on their motivated L2 learning grounded in multiple theoretical frameworks in the current L2 motivation research domain. Integrating findings from mixed methods research (both questionnaires and semi-structured interviews), the present study aims to gain a holistic and comprehensive understanding of what actually happens with English language learning motivation among the Chinese overseas students in a study abroad context. Based on the key variables identified from the students’ study abroad experiences, the present study highlights an integrated consideration of EFL education to improve learners’ motivation and enable them to better integrate themselves into the globalized world.

## Literature Review

### Research on L2 Motivation

#### Integrative and Instrumental Motivation

In the context of L2 or FL learning, motivation has been conceptualized in various perspectives such as Behaviorism, Cognitivism, Humanism, and Social Constructivism (Pishghadam et al., 2019a), in which motivation has been viewed as connecting people’s behaviors with external rewards (Staddon, 2001), internal drives (Maslow, 1943), the intrinsic/extrinsic attractiveness (Vroom, 1964) or people’s collective habits (Bourdieu, 1986) respectively. Over the past several decades, one of the dominant theoretical rationales in the mainstream motivational and educational psychology for L2 motivation research had been centered around language learners’ integrativeness/integrative motivation, the core concept of Gardner’s (1985, 2010, 2019) socio-educational model. Taking a social-psychological perspective, the theoretical concept reflects “a sincere and personal interest in the people and culture represented by the other group” (Gardner and Lamber, 1972, p. 132). The basic premise is that language learners’ willingness to become similar to the L2 group may sustain long-term motivation in L2 learning. However, the integrative concept has been challenged by L2 motivation researchers (e.g., Warden and Lin, 2000; Lamb, 2004; Dörnyei, 2005) in terms of its limitation in explaining L2 learners’ motivation in an ever-fluid global English learning environment that there is no specific target L2 ethnolinguistic group of the integration anymore. For example, in additional to attitudes toward members of the L2 community as a key antecedent of integrative motivation, Dörnyei et al. (2006) identified instrumentality/instrumental motivation (i.e., the pragmatic utility of learning the L2) in mediating L2 learners’ language choice and intended learning effort to study the L2. The instrumental motivation is conceived with both promotion focus (e.g., to learn English for enhancing one’s future professional career) and prevention focus (e.g., getting good grades in order not to disappoint one’s parents). The empirical evidence pointed out a need to reinterpret the theoretical concept from a different perspective for a better understanding of L2 learners’ motivation.

#### L2 Motivational Self System

By incorporating the possible selves theory of Markus and Nurius (1986) and Higgins’ (1987) self-regulation theory into L2 motivation research, the concept of the Ideal L2 Self and the Ought-to L2 Self were proposed as principal components in Dörnyei’s (2009) L2 Motivational Self System (L2MSS) to interpret a language learner’s motivation as an internal process of identification within the person’s self rather than social identification with an external ethnolinguistic group. The Ideal L2 Self refers to a desirable positive future image of oneself that a language learner would like to become; the Ought-to L2 Self reflects the self-image of what one should become to avoid possible negative outcomes or to meet expectations of important others. The core tenet is that when proficiency in the target language is part of one’s ideal or ought-to self, the person will be motivated dramatically to close the discrepancy between the current L2 self and possible L2 self (Dörnyei, 2009). The explanatory power of the ideal L2 self and the ought-to L2 self have been examined in an array of research (e.g., Csizér and Lukács, 2010; Kim and Kim, 2014; Al-Hoorie, 2018), although findings are not unequivocal, depending on different contexts and variables. A significant greater impact of the ideal L2 self (compared with the ought-to L2 self) on learners’ intended effort or motivated behavior in L2 learning has been reported in multiple studies (Magid, 2009; Kormos et al., 2011; Xie, 2014); the influence of the ought-to L2 self on effort has been revealed as insignificant (Kormos et al., 2011), or negative (Lee and Ahn, 2013), or positive but relatively low (Csizér and Kormos, 2009; Papi, 2010) in various studies.

#### International Posture

As a *lingua franca* in the physical and digital world, English is associated with globalized world-citizen identity (Dörnyei and Ryan, 2015). In addressing this broader form of identification, Yashima (2000) and Yashima et al. (2004) introduce the notion of international posture to refer to a generalized international outlook and a complex trait that includes “interest in foreign or international affairs, willingness to go overseas to stay or work, readiness to interact with intercultural partners and […] openness or a non-ethnocentric attitude toward different cultures” (Yashima, 2000, p. 57). In this sense, international posture captures a tendency to connect oneself to the international community rather than any particular target L2 group or culture. Specifically, international posture was operationalized to include four subcomponents: intergroup approach tendency, interest in international vocation and activities, interest in foreign affairs (Yashima et al., 2004), and “having things to communicate to the world” (Yashima, 2009, p. 156).

Most previous research has provided important insights in the motivating power of an imagined international community to learn L2s in foreign language learning contexts (Botes et al., 2020), particularly its role in promoting language learners’ willingness to communicate (WTC) in an L2 (L2 WTC; Yashima et al., 2004). L2 WTC was defined as “a readiness to enter into discourse, at a particular time with a specific person or persons, using L2” (MacIntyre et al., 1998, p. 547). If learners are conscious of how they relate themselves to the world around them (i.e., a higher level of international posture), they tend to actively engage in English-related actions because they probably generate possible selves communicating with international students and reading English language newspapers, and they might visualize their possible selves pursing an international career or working in a foreign country. Previous studies found that international posture is highly related to the ideal L2 self (Lee and Ahn, 2013; Xie, 2014; Kim and Lee, 2015), but is either weak or not related to the ought-to L2 self (Kormos et al., 2011; Lee and Ahn, 2013). It is argued that the “imagined international community” (Yashima, 2009) expanded the self by transcending time and space creating new images of the world and self (Wenger, 1998), and therefore, possible selves participating in an L2-using community can be sensed through the L2 self-involved in specific learning activities here and now.

#### Chinese Imperative

The concept relating to a learner’s current-state participation in an L2-using community is the L2 Learning Experience which concerns situated and executive motives referring to the immediate learning environment, and prior learning experience interacting with the current learning context (Dörnyei, 2009). This concept tends to take account of the impact of contextual factors in the surrounding learning environment on the individual learner’s language learning, and constitutes the bottom-up process for the two self-guides in Dörnyei’s (2009) L2MSS. Because of the inherently social nature of L2 acquisition, learning experience as a social practice is influenced by various social, economic, and cultural factors. Social motivation defined by Juvonen and Weiner (1994) involves the complex of motivational influences that stem from the sociocultural environment rather than from the individual, which indicates a situated construct and a dynamic facet of L2 motivation. In line with this, Ushioda (2009) proposes a person-in-context relational view to understand L2 learners as people who are located in particular culture and historical contexts, and points out that it is difficult to generalize L2 motivations that are embedded in specific settings due to the diversity of L2 learning contexts (Ushioda, 2013).

Prior studies have revealed the variety of learners’ L2 motivation in naturalistic-school settings (Ushioda, 2011; Henry and Cliffordson, 2017), rural–urban social contexts (You and Dörnyei, 2016; Li et al., 2018), and different nations (Taguchi et al., 2009; Yashima et al., 2017; Liu and Thompson, 2018; Farid and Lamb, 2020). For instance, studies indicated that the ought-to L2 self can be particularly significant in Asian countries such as Iran and China, where collectivism was dominant, and families tend to exert great impact on individuals’ motivation (Lockwood et al., 2005). In terms of the achievement, Zeynali et al. (2019) proposed the dichotomy of collective/individual motivation in their research to measure the significant influences of family, peers, and society on students’ motivation, and identified that the individualistic tendencies were more connected to students’ good educational performances compared to collective tendencies. The study was consistent with findings from previous research that the fulfillment of ideal L2 self is linked with higher achievement in language education (Rajab et al., 2012; Kim and Kim, 2014); whereas there is a negative association between Ought-to L2 self and achievement (Islam et al., 2013; Subekti, 2018). English education inevitably conveys ideology, beliefs, values, customs, or in a nutshell “culture” (Pishghadam et al., 2015), and consequently requires researchers to consider different populations within heterogeneous contexts when exploring their L2 motivation.

For Chinese students, English language learning is compulsory at all levels of the education system. Knowledge of English is essential for Chinese students to perform well in high-stakes exams for attaining academic success as well as advancing in both competitive local and global job markets. In the Confucian meritocracy sociocultural and educational context, through the socialization process, one is expected to fulfill one’s identification with obligations of the social role by achieving goals that are highly valued by the society for the sake of maintaining a harmonious social network (Yu and Yang, 1994; Chen et al., 2009; Hwang, 2012). Chinese students, therefore, are considered to have successfully fulfilled their role obligations if they can accomplish academic success (including English language leaning). In addition, the Confucian relationship is consolidated in such a way that the person’s success is not only a positive reflection on the individual but also on families and clans. Previous studies of achievement motivation have revealed the internalization of socially oriented motivation and the decisive impact on forming language learners’ effort in Confucian-influenced contexts (Li, 2004; King et al., 2012; Cheng and Lam, 2013).

Current Chinese students’ EFL motivation studies display varied results. Required motivation was noted to show that Chinese students appeared to be motivated by requirements rather than any interest in integration or any clear instrumental orientation yield (Warden, 2000; Warden and Lin, 2000). Chen et al. (2005) expand this concept further by proposing the “Chinese imperative” in describing a motivator that is valued by individuals in a Chinese cultural study context, which is more than the general labels of collectivism and even requirements. The construct reflects an emphasis on requirements that are internalized within the Chinese cultural study context. Wei’s (2007) study found that Chinese students had higher instrumental than integrative motivation, and Cai (2010) concluded that many Chinese college students lack motivation for learning English due to the mismatch between their relatively low achievement and considerable time they spend on English learning. The influence of familial and social obligations on learners’ strength of ought-to self in the Chinese context has been emphasized in Magid’s (2009) study; on the contrary, positive ideal self-images of using English, positive attitudes toward studying English, construction of native speaker-based images for the ideal L2 self, and the anti-ought-to self in the face of challenges among Chinese students in their English learning have been uncovered in other empirical studies (Gao, 2010; Zheng, 2013; Thompson and Vásquez, 2015; You and Dörnyei, 2016). The inconsistent results point to a need for more research to further understand Chinese students’ English language learning motivation in specific learning contexts.

#### Active/Passive L2 Motivation

By and large, the foregoing views are in line with the ideas that different cultural and linguistic contexts can be vital in constructing peoples’ values, knowledge and practices (Piaget, 1954; Whorf, 1956; Vygotsky, 1978), and experiences give rise to individuals’ ultimate knowledge of the world (Matthews, 1992), including motivation on language learning. Pishghadam et al. (2015) adopted a broader view, i.e., sensory relativism, to underscore the role of senses in relativizing people’s understanding of the world. They argued that sensory experiences such as hearing, seeing, or touching can contribute to the development of different emotions which, in turn, shape people’s perception of the world. The grounded core concept of this view is emotioncy which refers to the varying emotion levels evoked by senses (Pishghadam et al., 2013a,b). Pishghadam et al. (2015) pointed out that people might employ various kinds and degrees of emotions toward different concepts in a language based on their sensory experiences. To further explicate, Pishghadam (2015) devised an emotioncy-matrix labeled with different types and measures of emotioncy. The emotioncy-matric includes six-levels originally: Null emotioncy (0, Not familiar), Auditory emotioncy (1, Heard), Visual emotioncy (2, Hear and Seen), Kinesthetic emotioncy (3, Hear, Seen, and Touched), Inner emotioncy (4, Heard, Seen, Touched, and Used), and Arch emotioncy (5, Heard, Seen, Touched, Used, and Done research on), and was later extended by adding a new level, Mastery, to reflect individual being able to develop and produce content (Heard, Seen, Touched, Used, Done research on, and Created; Pishghadam et al., 2019b; Miri and Pishghadam, 2021).

In L2 learning, individuals may change their emotioncy levels forward and backward along with the emotioncy continuum levels according to various social, economic and cultural factors in the language education environment. For instance, a learner participating in a class can move from avolvement (null level) to exvolvement (auditory, visual, and kinesthetic levels) and eventually to involvement (inner and arch levels) or metavolvment (mastery level) through teachers’ actions in certain ways. In this sense, emotioncy that encompasses emotions, senses and frequencies, can be understood as “sensory capital” that deals with the amount of sensory access one has, for language learners, and teachers are considered as “envolvers” who can affect and help learners to increase their emotioncy levels in the classroom, so that consequently change their sensory capitals and understanding toward a concept in an L2 (Pishghadam et al., 2019c; Pishghadam and Shakeebaee, 2020). Previous studies on emotioncy have demonstrated the role of sensory emotioncy types in the second language acquisition. For example, Makiabadi et al. (2019) identified a significant and positive correlation between all three types of sensory emotioncy (emotional, cognitive, and sociocultural) and language learners’ WTC, which is consistent with prior studies that revealed a significant positive association between learners’ emotioncy and their WTC (Pishghadam, 2016).

In the realm of L2 motivation specifically, Pishghadam et al. (2019a) introduced the dual continuum model of motivation based on the concept of emotioncy. The model consists of engagement and involvement as two fundamental constructs, the former refers to full attention and concentration of individuals at the time of activity performance (Rothbard, 2001); rooted in the concept of emotioncy, the latter deals with individuals directly experiencing or doing research on something to get additional information. In other words, engagement and involvement are tied to thinking (mental activity) and doing (physical activity) respectively. The presence or absence of engagement is interrelated with various degrees of sensory involvement that divides “the model into two halves (i.e., active and passive) and four slices (comprising active motivation, active demotivation, passive motivation, and passive demotivation)” (Pishghadam et al., 2019a, p. 5). According to Pishghadam et al. (2019a), active motivation is when an individual is fully engaged and involved in performing something; it moves to be active demotivation when the performance becomes a mechanical one due to the lack of mental engagement; passive motivation concerns individuals have not been able to turn motivational preferences into action; and finally, passive demotivation pertains to represent no cognitive or physical activity regarding an activity. The unveiling of different sides of motivation is meaningful in the realm of both language education and teacher education. For example, Zeynali et al. (2019) study illustrates that motivating demotivation construct is the most important variable affecting students’ achievement at the academic level, and points out a need for students to develop perceptions about their abilities to perform in new, challenging, or even unwanted environment (such as a study abroad context). Accordingly, as envolvees, students’ active motivation for foreign language learning can be increased through teachers’ emotioncy, e.g., stroking behaviors that refers to positive teacher interpersonal communication behaviors, in their teaching practices (Pishghadam et al., 2021).

### L2 Motivation in Context of Study Abroad

It is becoming more and more accessible for university students in most education contexts worldwide to approach study abroad opportunities (Pérez-Vidal, 2015; Jackson and Oguro, 2017). English language learners from China account for a large proportion of international university students. It is pertinent that individual difference factors (e.g., L2 motivation) of learners can mediate study abroad experiences and to what extent the learners can acquire envisioned linguistic benefits from the experience (Pérez-Vidal, 2017). For Chinese students, a transition from an EFL learning context with Confucian traditions to an ESL context with new sociocultural norms and values means more opportunities as well as challenges in L2 learning, i.e., the root of the major problem in successful living and academic success. Although motivation has been investigated frequently in the study abroad context, most relevant studies are grounded in Gardner’s (1985) framework: for example, Hernández (2010) investigate the impact of integrative motivation and interaction with the Spanish culture on enhancing learners’ speaking performance; Sasaki (2011) identifies the role of intrinsic motivation in improving Japanese students’ writing skills, and willingness to seek opportunities for target language use has been identified as significant in elucidating Spanish/Catalan leaners’ language gains by Llanes et al. (2015). As argued by Pawlak et al. (2020), few studies have been able to keep up with the latest developments in motivation research in Second Language Acquisition, except that Larson-Hall and Dewey (2012) explore the function of a number of motivational variables, including the ideal L2 self and L2 learning experience and aptitude, on L2 learners’ proficiency in Japanese learning.

More recently, researchers tend to address more complex L2 motivational experiences in study abroad context. For instance, Fryer and Roger’s (2018) study reveals participants’ ability to sustain future imagined selves and display motivation learning behaviors by using semi-structured interviews and a narrative journal with eight Japanese students. Yu et al. (2018) document the transformation from the ideal to the “dreaded” L2 self (or vice versa) among Chinese PhD students studying in New Zealand. By using mixed methods research, Du (2019) reports that study abroad experiences have a profound impact on the language learners’ ideal self-images. To capture the dynamic and complex nature of motivation, Fukui and Yashima (2021) explore how two Japanese students’ ideal and ought-to second- and third-language selves emerged along with an ideal multilingual self in one of the students while abroad.

In summary, most previous studies on Chinse students’ L2 motivation were conducted in the EFL Chinese sociocultural context in main. Among them, L2 motivation has been investigated in their own right with focus on some isolated motivational variables (e.g., ideal L2 self or ought-to L2 self) at the expense of meaningful others (e.g., international posture, Chinese imperative), and few attempts to embrace a combination of multiple significant motivational constructs and examine their complex impacts on language learners’ L2 learning. In this sense, Chinese overseas student’s motivation on English language learning in a study abroad context remains fragmentary understanding.

Accordingly, the current study endeavors to address the above gaps by posing the following research questions:

1. How are motivation variables associated with the Chinese overseas university students’ L2 learning behaviors?
2. How do the students perceive the role of different motivational variables in their L2 learning in the United Kingdom?
3. What are the key motivational variables influencing the students’ motivated L2 learning in study abroad?

## Methodology

### Research Context and Participants

The current study was conducted in the United Kingdom, where international students with multicultural backgrounds and local native English speakers constitute the main context for Chinese overseas students’ living and education. The current study involved 217 participants aged 22 to 23 years old. They were accessed through an online Chinese social network to take part in the online survey, and nine of them were willing to participate in interviews. Participants attended different university institutions around the United Kingdom, and all of them were taking master degree programs with a range of majors (including arts and law; engineering and physical science; business, finance and economics; education, and culture and media) at the time of the study. Participants shared common prior educational experiences in mainland China, where English was taught as a compulsory subject from primary to higher education.

### Data Collection and Instruments

As an inter-discourse (quantitative and qualitative) methodology rather than a combination of separate “methods,” mixed methods research allows researchers to integrate the advantages of both research approaches in making more rigorous and insightful inferences about applied linguistic research problems (Riazi, 2016). Coordination of multiple theoretical perspectives and an integration of different sorts and facets of data are encouraged in order to collectively enrich understanding of second language development issues in the real world (Douglas Fir Group, 2016; Toth, 2019). In the domain of L2 motivation research, a shift in research orientation with a growth of mixed methods studies in recent years has contributed to a revitalisation of the research environment (see Boo et al., 2015), and also has shed much light on the great potential in addressing critical L2 motivation research questions.

In the current study, the questionnaire was designed according to principles and procedures suggested in Dörnyei (2003) to collect data for examining associations between multiple motivational variables and Chinese overseas students’ L2 motivated learning behaviors. The complete questionnaire consists of a total of 55 five-point Likert scales items, 21 five-point numerical rating scales items and four short questions for participants’ demographic data. The scales were adopted from the Dörnyei et al. (2006) Hungarian studies (i.e., instrumentality, attitudes toward L2 speakers/community, cultural interest), the L2 Motivational Self System (ideal L2 self, ought-to L2 self), and other relevant important theories (e.g., international posture, parent encouragement, exam passing, WTC). Most of the items for the scales come from previous validated questionnaires of relevant literature in Dörnyei and Ushioda (2009) (see more details in Table 1). Additionally, some of them were adapted and newly designed to fit in with the specific research context of Chinese overseas university students in the United Kingdom For example, the item “I often imagine myself as someone who is able to speak English” was modified to “I often imagine myself as someone who is able to speak English like native speakers” by specifying the level of proficiency as the participants had already learned to speak English, and the item “I would like to work in China after I graduate in the U.K.” was designed particularly for Chinese overseas learners to measure the interest in international vocation. Before being generated online, the questionnaire was translated into Mandarin and was tested to identify any ambiguous items or instructions that needed clarification.

**Table 1:** A summary of definition, item reference, sample item and number of items of motivational concepts that the questionnaire intended to measure.

	Motivational concepts	Definition	Item reference, sample item, and number of items
Motivational variables	Ideal L2 self (ILS)	It refers to the L2-specific facet of one’s ideal self	Ryan (2009): e.g., I can imagine speaking English fluently with international friends. (*N* = 4)
	Ought-to L2 self (OLS)	It concerns the attributes that one believes one ought to possess (i.e., various duties, obligations, or responsibilities) in order to avoid possible negative outcomes	Taguchi et al. (2009): e.g., If I fail to learn English, I will be letting other people down. (*N* = 6)
	Instrumentality (Ins)	Measuring the pragmatic utility of learning English.	Taguchi et al. (2009): e.g., Studying English is important to me because English proficiency is necessary for promotion in the future. (*N* = 9)
	Parental encouragement (PEn)	Examining the extent of parental roles in influencing their children’s learning English either active or passive.	Chen et al. (2005): e.g., My parents encourage me to take every opportunity to use my English (e.g., speaking and reading). (*N* = 8)
	Exam passing (EP)	Measuring the regulation of duties and obligations in passing exams.	Chen et al. (2005): e.g., I have to study English because I do not want to fail the course. (*N* = 6)
	International posture (IP)	It concerns learners’ attitudes toward English as an international language.	Yashima (2009): e.g., I want to make friends with international students studying in the UK. (*N* = 10)
	Attitudes to L2 speakers/community and culture (ASCC)	It investigates students’ attitudes toward the people, community of the target language and the interest in the cultural products of the L2 culture.	Taguchi et al. (2009): e.g., Would you like to know more about people from English-speaking countries? (*N* = 8)
Motivated Learning behaviors outside the classroom	L2 willingness to communicate (WTC)	It measures the learner’s willingness to enter into discourse using English at a particular situation with a specific person or persons.	Yashima (2009): e.g., When you find an acquaintance standing before you in a line. (*N* = 8)
	Frequency of communication (FC)	Measuring the regularity of the student’s using English in their communication.	Yashima (2009): Do you talk to international students or teachers in English outside the class? (*N* = 5)
	Intended learning effort (ILE)	Referring the strength or intensity of individuals’ visions of themselves as users of English.	Ryan (2009): e.g., I am working hard at learning English outside class. (*N* = 9)

Moreover, semi-structured interviews were conducted with the nine participants in order to further explore potential motivational variables in relation to the students’ L2 learning in reality from a “person-in-context relational” (Ushioda, 2009) perspective. The participants were required to consider any subjects, objects, situations, and contexts that they perceived as significant in motivating their English language learning during the time of research. The following interview questions were prepared in advance as a general guideline to encourage the participants to give more information in the interview. More specific newly created questions also were asked during the interview to further explore the potential links between incentives and relevant learning behaviors. All interviews were conducted in Mandarin (the participants’ mother tongue) in about 90 min. All interviews were digitally recorded and transcribed either verbatim or partially for further scrutiny.

1. Can you give a detailed description about a situation that you felt motivated in learning or using English?
2. What and why do you think it is significant in motivating your English language learning?
3. How does it motivate you?
4. What did you do regarding English language learning when you are motivated?
5. Did you or are you approaching what you want to attain?
6. If not yet, why do you think this is the case/what do you think may be the problem?
7. What would you like to do next in approaching your possible goals or desires?

### Data Analysis

The original questionnaire data was processed using SPSS 23.0, STATA 17.0, and AMOS 18.0. The Structural Equation Modeling (SEM) model was adopted in order to produce more robust results. SEM provides a dependable framework that enables researchers to analyze not only the associations between variables but also in what ways (i.e., path analysis) latent variables relate with observed variables. The assessment of the model was based on five criteria: measure of sampling adequacy, indicator reliability, construct reliability, convergent validity, and discriminant validity (see Tables 2, 3). To validate the SEM model specification, variables (questions) with factor loadings below 0.7 were eliminated. The KMO & Bartlett test indicates that the construction of items is accepted for a SEM analysis and the values of construct reliability and Average Variance Extracted (AVE) were all above 0.7 and 0.5 respectively, indicating that observed variables refined in this study well reflect proposed latent variables (Chin, 1998). In addition, the square roots of AVEs were larger than the correlations between constructs. This result suggests a satisfactory discriminant validity (as presented in Table 3).

**Table 2:** SEM model analysis results.

Components		KMO	Factor loadings	Construct reliability	AVE
Ideal L2 self (ILS)	ILS1: Whenever I think of my future career, I imagine myself being able to use English	0.663	0.809	0.833	0.625
	ILS4: I can imagine speaking English fluently with international students		0.748		
	ILS5: When I think about my future, it is important that I use English		0.812		
Ought-to L2 self (OLS)	OLS2: My parents /family believes that I must study English to be an educated person	0.771	0.708	0.850	0.587
	OLS 4: Studying English is important to me because an educated person is supposed to be able to speak English		0.861		
	OLS 5: When I think about my future, it is important that I use English		0.758		
	OLS 6: If I fail to learn English, I will be letting other people down		0.728		
Instrumentality (Ins)	Ins3: Studying English is important to me because English proficiency is necessary for promotion in the future	0.689	0.807	0.854	0.662
	Ins7: Studying English is important to me in order to attain a higher social respect		0.826		
	Ins2: Studying English is important because with a high level of English proficiency I will be able to make a lot of money		0.807		
Parental encouragement (PEn)	PEn2: My parents encourage me to attend extra English classes after class (e.g., free English classes)	0.668	0.760	0.860	0.671
	PEn6: Being successful in English is important to me so that I can please my parents/relatives		0.860		
	PEn8: I have to study English, because, if I do not do it, my parents will be disappointed with me		0.835		
Exam passing (EP)	PEx2: I have to learn English because I do not want to fail the English course	0.650	0.817	0.848	0.651
	PEx4: Studying English is important to me in order to bring honor to my family		0.859		
	PEx6: Studying English is important to me, because I would feel ashamed if I got bad grades in English		0.739		
International posture (IP)	IP3: I would like talk to international students in the class	0.814	0.752	0.902	0.650
	IP8: I often talk about situations and events in foreign countries with my family and/or friends		0.857		
	IP10: I have thoughts that I want to share with people from other parts of the world		0.831		
	IP11: I have ideas about international issues, such as environmental issues and north–south issues		0.822		
	IP6: I’m interested in an international career		0.763		
Attitudes to L2 speakers/community and culture (ASCC)	ASLL1: Do you like the music of English-speaking countries (e.g., pop music)?	0.811	0.732	0.860	0.545
	ASLL2: Do you like English films?		0.735		
	ASLL3: Do you like English magazines, newspapers, or books?		0.722		
	ASLL5: Do you like to travel to English-speaking countries?		0.749		
	ASLL7: Do you like meeting people from English-speaking countries?		0.753		
Willingness to communicate (WTC)	WTC2: When you have a chance to talk in a small group of strangers	0.759	0.840	0.855	0.598
	WTC3: When you are given a chance to talk freely among a group of acquaintance		0.792		
	WTC5: When you together with a small group of friends		0.751		
	WTC1: When you find an acquaintance standing before you in a line		0.703		
Frequency of communication (FC)	FC1: Do you talk to international students or teachers in English outside the class?	0.820	0.793	0.900	0.692
	FC2: Do you try to talk during outside classroom activities such as parties?		0.849		
	FC4: Do you talk with English-speaking friends or acquaintance in English outside class?		0.835		
	FC5: Do you try to talk when you had a chance to speak English outside class?		0.848		
Intended learning efforts (ILE)	ILE1: I am working hard at learning English outside class	0.703	0.783	0.840	0.568
	ILE2: It is extremely important for me to learn English		0.717		
	ILE5: I can honestly say that I am really doing my best to learn English		0.798		
	ILE9: I have an intention to accumulate vocabulary in daily life, either when I read newspapers or go shopping		0.714		

**Table 3:** Discriminant validity test.

	ILS	OLS	Ins	PEn	EP	IP	ASCC	WTC	FC	ILE
ILS	0.791									
OLS	0.378	0.766								
Ins	0.508	0.741	0.813							
PEn	0.093	0.545	0.333	0.819						
EP	0.072	0.444	0.416	0.516	0.807					
IP	0.375	0.276	0.280	0.016	0.067	0.806				
ASCC	0.251	0.172	0.170	0.037	0.181	0.501	0.738			
WTC	0.514	0.283	0.386	0.091	0.216	0.567	0.307	0.773		
FC	0.438	0.271	0.281	0.035	0.013	0.532	0.273	0.496	0.832	
ILE	0.311	0.316	0.385	0.265	0.262	0.517	0.290	0.439	0.292	0.754

Coefficients on the diagonal (in boldface) are the square root of the average variance extracted (AVE). Other coefficients are correlations between constructs.

The goodness-of-fit indices indicate how well the model being analyzed explains the current dataset. A range of tests were included, as there is not a universal benchmark to determine an absolute level of “goodness-of-fit” and each test method has its own deficits. Therefore, both absolute fit indices and incremental fit indices were included. As can be seen in Table 4, all indices were at satisfactory levels, suggesting that the final model being analyzed is acceptable.

**Table 4:** Goodness-of-fit indices for the final model.

Reference standards	WTC	FC	ILE
CMIN/df < 3 (Carmines and McIver, 1981)	1.967	2.002	1.899
RMSEA<0.06 (Hair et al., 2006).	0.061	0.055	0.058
SRMR<0.08 (Hair et al., 2006).	0.077	0.073	0.071
CFI > 0.9 (Bentler and Bonett, 1980)	0.943	0.987	0.903
TLI > 0.9 (Bentler and Bonett, 1980)	0.969	0.955	0.924

In addition, the qualitative analysis software NVivo 12 was employed for analyzing data collected from the interviews in order to answer the second research question. Open coding was conducted initially through repeated reading, constant interpreting, and reinterpreting the transcripts (Bazeley and Jackson, 2013), which allows obtaining a broad suite of codes, such as “local cultural interest,,” “Western fashion interest,,” “English as an international communication tool,” “international friend,” and “intended listening.” Then, proceeding with a more analytical approach (Richards, 2021), data cross datasets were revisited critically, and potential links between these initial codes were identified, for example, the relationship between “local cultural interest” and “intended listening” was identified after further interrogation of the data and reflections. Subsequently, the codes were grouped in different categories on the basis of the multiple motivational variables and motivated behaviors employed in the quantitative study. At this stage, participants’ perceptions of the roles of motivational variables in relation to their motivated L2 learning were clearly apparent, for instance, the role of international posture in promoting L2 WTC and the role of parental encouragement in facilitating intended learning effort. The findings obtained from the interviews were then integrated and compared with the results of the questionnaire. Through this analytical inquiry process, answers for the third research question were clarified.

## Results

### Associations Between Motivational Variables and L2 Learning

**Figure 1:**
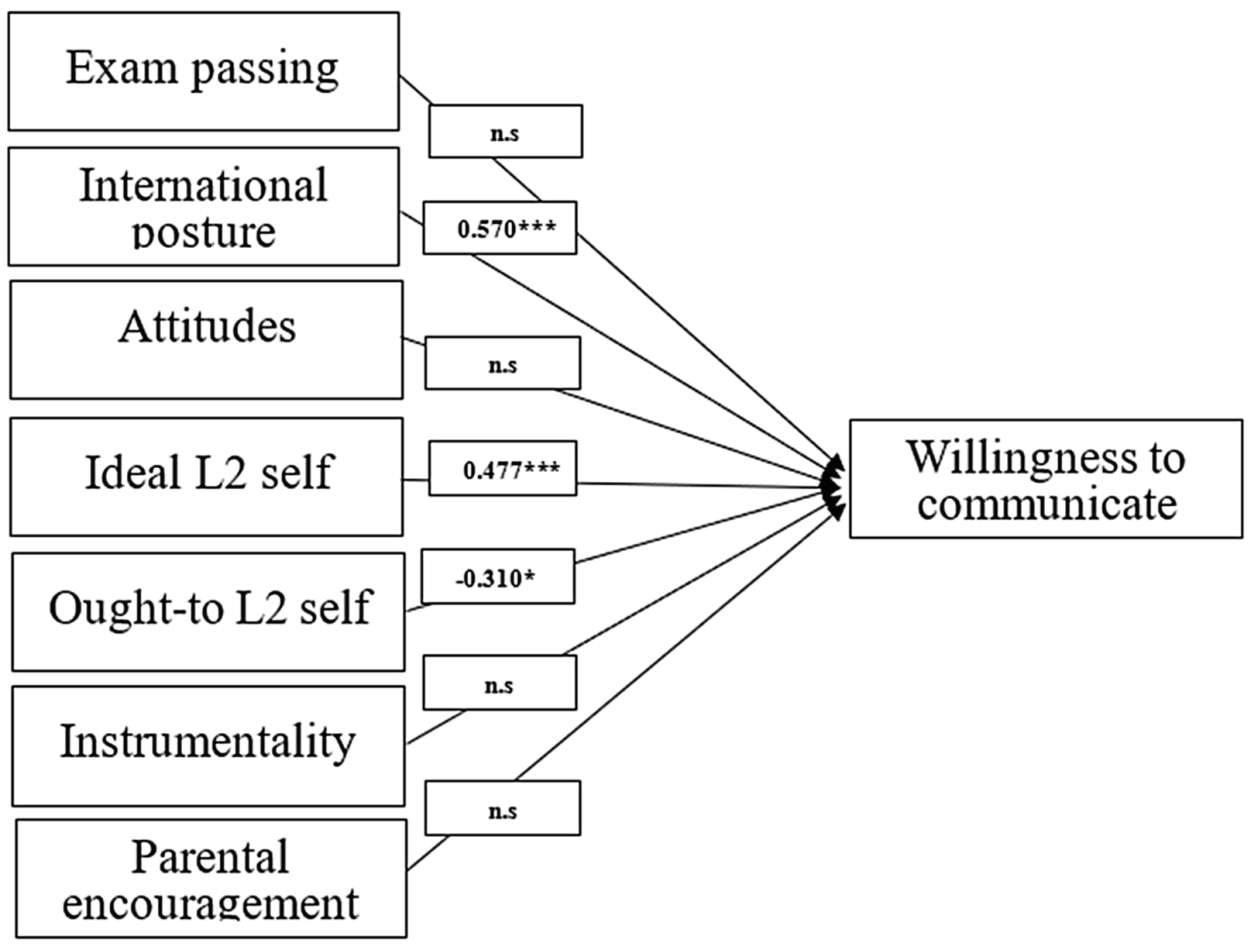
Relationships between motivational variables and willingness to communicate.

SEM analysis indicates that motivational variables have correlations of various strength with the Chinese overseas students’ different motivated English language learning behaviors. Concerning the effects of proposed variables on the level of WTC, as can be seen in Figure 1, it appears that international posture had the strongest positive effect (0.570), followed by ideal L2 self (0.477). In comparison, ought-to L2 self-exhibited a significant negative effect on WTC (−0.310), and attitudes to L2 Speakers/community and culture, instrumentality, parental encouragement, and exam passing did not show any obvious influence on language learners’ L2 WTC.

**Figure 2:**
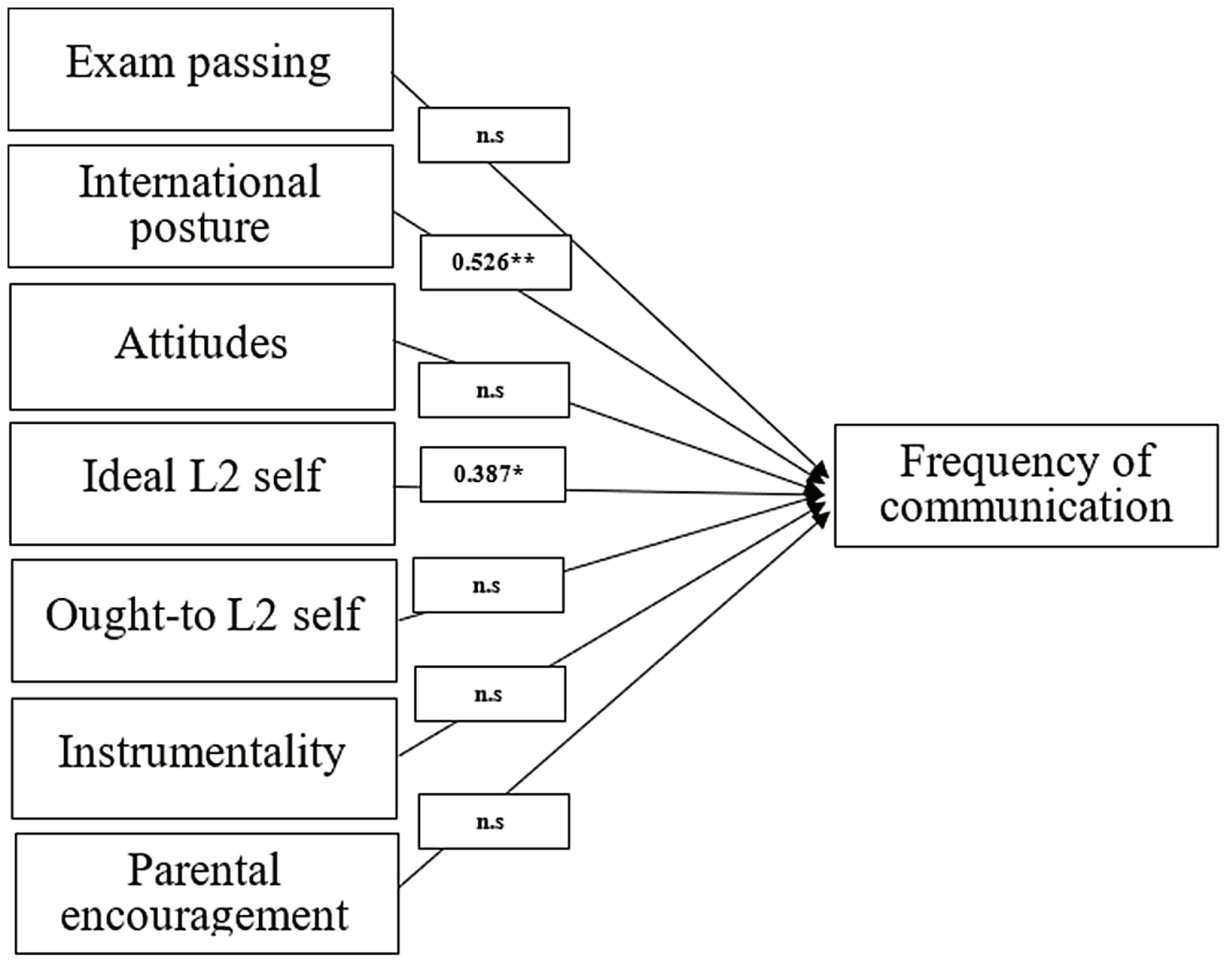
Relationships between motivational variables and frequency of communication.

Regarding frequency of communication (Figure 2), international posture (0.526) and ideal L2 self (0.387) had statistically significant positive impacts, and the remaining variables seem to be non-significant in relation to the learners’ frequency of communication.

**Figure 3:**
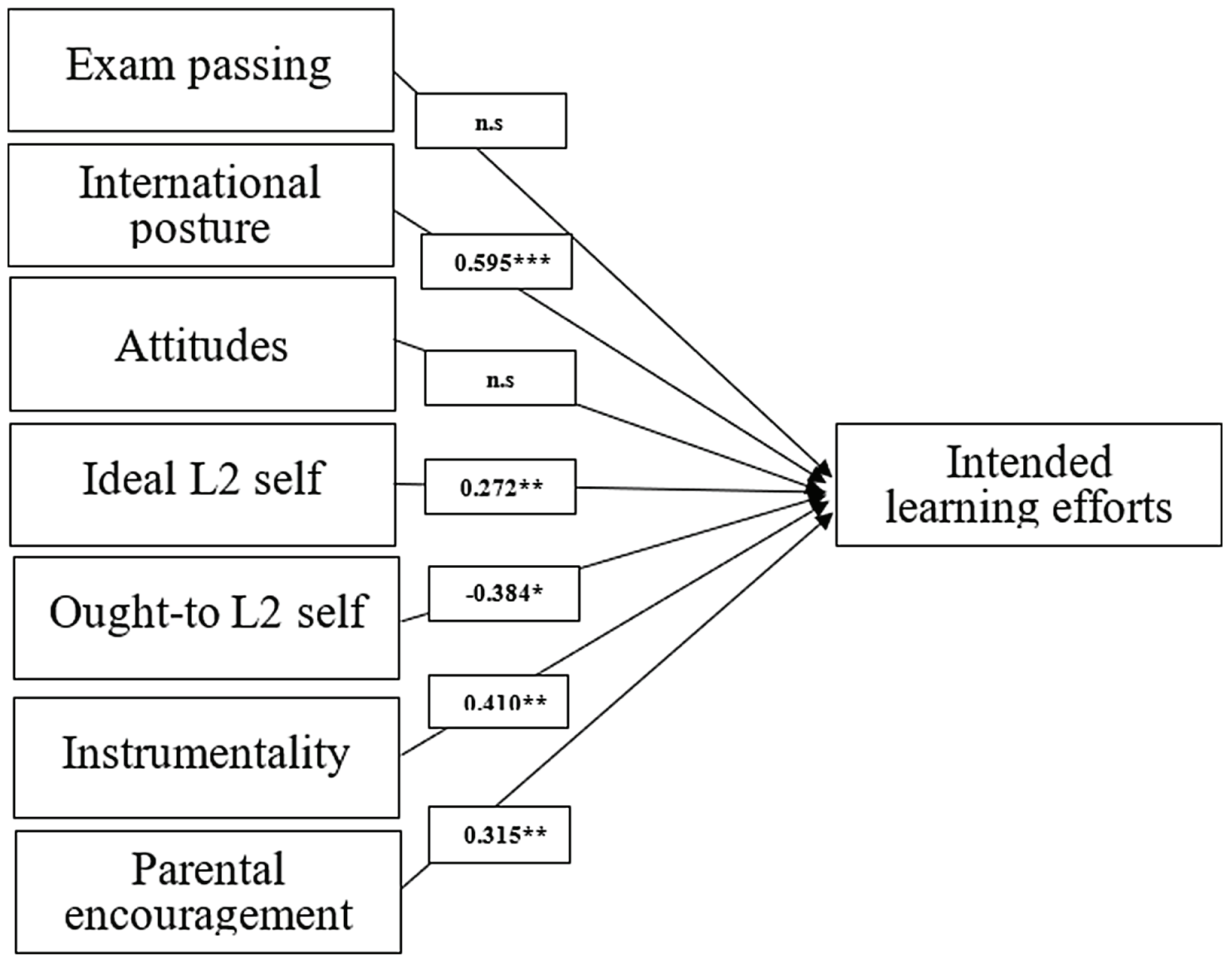
Relationships between motivational variables and intended learning efforts.

Moreover, consistent with the above findings, the model specification of intended learning efforts in Figure 3 revealed that the effect of international posture again is the highest, with a coefficient of 0.595, followed by instrumentality (0.410), parental encouragement (0.315), and ideal L2 self (0.272); the effect of ought-to L2 self is significantly negative (−0.384). Attitudes to L2 speakers/community and culture, and exam passing did not have prominent effect on intended learning efforts.

### Learners’ Perception of the Role of Motivational Variables in Their L2 Learning

#### The Pivotal Role of International Posture and Ideal L2 Self in L2 Learning

In accordance with the results from quantitative analysis, data from participants’ interviews also indicates the significant roles of international posture and ideal L2 self on all sorts of language learners’ L2 motivated learning behaviors, particularly the impact of international posture in guiding the students’ WTC and frequency of communication. For example, in reflecting on her L2 communication experience during traveling, Emma mentioned that.

“At the beginning, I’m very shy to ask ways or ask for help, because I’m afraid I do not speak English fluently and lack of vocabulary. After traveling for few days, I started to realize that it is not necessary to worry about it, as English is a means for communication, particularly in such an environment that English is the only common language. I’m happy that I can communicate successfully with French and Spanish people who can use very limited English words. Although sometimes we tried to use simpler words to negotiate meanings, but I think it is English that connects me with other people in the global world, and now I feel more confident and more willing to communicate with others in English. I also invested more in learning English, so that 1 day I can communicate more fluently with people from all over the world.”

The above extract shows how international posture as perceived by Emma motivated her WTC and frequency of communication in English during as well as after the journey. Additionally, Emma mentioned in the interview that how she prepared herself for traveling including reading materials in English about the scenic spots, learning new words for understanding the cultures and histories relevant to the scenic spots in foreign countries, and writing travel journals and sharing her travel experiences on Facebook as a way of practicing English. It seems evident that Emma’s international posture, especially the knowledge orientation with interest in international news and her vision of ideal L2 self (i.e., communicate more fluently with people from other countries) worked as an impetus in guiding her intended learning effort. This confirms the operation of international posture in interest in foreign affairs (Yashima et al., 2004) and having things to communicate to the global world (Yashima, 2009). Additionally, from the perspective of emotioncy, it can be argued that international posture enables Emma to shift from exvolvement (i.e., merely heard and seen the vocabularies) to involvement (i.e., using words and participating in communication) or even metavolvement (i.e., creating travel journals in English on Facebook).

Moreover, the impact of international posture, particularly the intercultural friendship orientation, on learners’ L2 communication was identified by other interview participants, for example:

“I’m very glad that we can make a friend with the landlady of this small restaurant in Germany. Although she did not speak English very well, I still can feel her enthusiasm and warm welcome to the guests worldwide” (Ida).

“… fashion is an eternal topic for girls. I’d like to learn something about fashion in English, so that I can get into the group and make friends with them. For example, I made friends with a French girl as we have our common interest about making up” (Vivian).

“Chinese take away, is more like a way to exchange cultures and get involved in the intercultural activities in the local community. I’m very excited and motivated to introduce Chinese traditional food to my friend…” (Kevin).

These excerpts revealed the students’ passion and enjoyment in developing international friendships with people in the global community. Their active involvement in social interactions with non-Chinese people and openness to local people reflect their ideal L2 selves of future English-using participants in an international community. To some extent, the finding indicates that peers in the global community can work as important envolvers in enriching language learners’ sensory experiences, and therefore encourage more active motivation in English language learning.

The data suggests that learners’ view about the global significance of English does not only work as a highly important driving force in L2 learning, but also contributes to students’ future image of ideal selves as successful English language users in the global community. To some extent, it can be said that international posture tends to be an internalized motive that significantly related to the Chinese overseas students’ L2 self-concept rather than a reference with extrinsic factors. The findings provide convincing support for this assumption, and at the same time, conform with similar results found in other Asian and European settings in previous investigations about international posture (Lamb, 2004; Kormos et al., 2011; Xie, 2014; Kim and Lee, 2015).

#### Instrumentality and Parental Encouragement in Guiding L2 Intended Learning Effort

Data from qualitative analysis also points out the important role of instrumentality in relation to language learners’ L2 intended learning effort. Both promotional and preventional impacts in instrumentality can be identified across interview participants’ datasets, which offered a more nuanced understanding that complemented the results of the quantitative study. The promotional aspect in instrumentality in stimulating the students’ intended effort regarding English language learning can be reflected in meeting personal demands in daily life in the United Kingdom and future career demands in pursuing a job in the global community. For instance, Joanna stated in the interview that.

“Since more vocabularies learned from reading magazines, I became more willing to communicate with the salesperson when I go shopping, as I can express my personal needs more clearly, and we both can understand each other more effectively.”

As can be seen, the excerpt explained a possible relationship between Joanna’s invested effort in learning new words from reading magazines and her instrumental orientation of being able to express personal needs more effectively while living abroad. This seems in accord with a prior study that identified that high proficiency in English was more important for language learners studying abroad to achieve their personal goals, which therefore made them more likely to invest effort in learning English (Li, 2014).

Additionally, data from participant Leo also supports this finding but with an emphasis on a close link between orientation of obtaining a professional job in the future and his intended learning efforts, as shown in the following excerpt:

“I perceive English as a great advantage in obtaining a good job after I go back to China, so I paid attention to accumulating terminology and knowledge in the field of psychology. I want to become an expert in this domain, and I think I will keep updating my knowledge with the cutting-edge research worldwide by reading relevant publications in English.”

It appears that English changed from being the core content of learning in China to being an instrument for core content learning in the United Kingdom as well as a key factor in becoming professionally successful in the future, considering the change of context of English as a *lingua franca*. In this case, the promotional instrumental motive impels the learner to spend considerable time and effort in vocabulary learning and academic reading in English in moving toward becoming an expert in the specific domain. It also indicates that instrumentality can be a strong impetus when the ideal images are associated with being professionally successful. The link between intended effort in learning English and desire for future career seems evident across the interview participants in this study who either want to find a job in the United Kingdom or in other countries, or who prefer to go back to China after they graduate. To a certain extent, this finding is consistent with Li’s (2014) study that in comparison with the EFL learners, promotional instrumentality was a more powerful motivator of learning behaviors for the ESL learners, as they were more eager to become academically or professionally successful since they invested more in studying abroad. At the same time, it is worthy to note that compared to the role of international posture, the promotional instrumentality seems to work well in retaining language learners’ active demotivation or passive motivation (i.e., intended learning effort) with a relative lower emotioncy level (e.g., learning vocabulary or reading academic articles).

Meanwhile, the preventional dimension in instrumentality was also perceived by the interview participants in regulating their L2 intended learning effort to avoid the presence of negative outcomes. For instance, some participants explained their intention “to accumulate professional vocabulary as much as possible,” otherwise they “cannot understand lectures and participate in group discussions.” In addition, the significance of devoting effort to academic reading and writing in determining the success of completing assignments and passing exams was also mentioned. In this case, it can be said that instrumental prevention worked as an ought-to self-guide in motivating the learners’ L2 learning efforts, which is also reflected in a significant correlation between instrumentality and ought-to L2 self (=0.741) shown in the quantitative analysis. The finding is consistent with You and Dörnyei’s (2016) study that confirmed that the desire to avoid academic failure could be a powerful factor influencing motivation of Chinese learners of English. Moreover, distinct from the widely held belief that Chinese learners are primarily instrumentally motivated (see You and Dörnyei, 2016), results from the current study suggest a complex operation of promotional and preventional as well as specific contextual variational aspects of instrumentality in mediating the Chinese overseas students’ intended effort in English learning.

Moreover, parents’ encouragement was considered by some interview participants as influential in motivating their intended effort in learning L2. For instance, Sarah highlighted her parents’ encouragement in requiring her to “acquire a large quantity of vocabulary” and “communicate frequently and fluently in English, or at least do not feel nervous in speaking English.” This encouragement was perceived as an impetus in motivating her to accumulate words from various resources in her daily life and communicate with non-Chinese speakers as much as possible, such as sharing her learning experience with English-speaking flatmates and participating in social activities in her local community. This offered more detailed evidence in supporting the prominent correlation shown between parental encouragement and intended learning efforts from quantitative analysis, and highlighted the role of individuals’ parents as envolvers in expanding potential opportunities for language learners’ sensory experiences.

### The Key Motivational Variables Influencing Language Learners’ L2 Learning During Study Abroad

Integrating the results from both quantitative and qualitative analysis, international posture was identified as the most significant factor in motivating the Chinese overseas university students’ English language learning. At the same time, the ideal L2 self was indicated as a key impetus in determining their motivated L2 learning behaviors. Instrumentality and parental encouragement were also shown to be important motivators in guiding their L2 learning, especially their intended learning efforts in the context of study abroad. However, what is inconsistent in the findings is that ought-to L2 self tended to be an influential factor in demotivating many of the learners’ L2 learning behaviors in quantitative analysis, whereas it was not evident in participants’ interview datasets. A more detailed discussion follows.

## Discussion and Implication

### International Posture as a Crucial Motivator for the Chinese Overseas Students’ L2 Learning

International posture is the most prominent motivational variable that has significant correlations with all three kinds of motivated behaviors, including L2 WTC, frequency of communication and intended learning effort. The results coincide with findings reported in prior research (e.g., Yashima, 2009; Kormos et al., 2011; Xie, 2014; Chen et al., 2020) and reveal that Chinese overseas students studying in the United Kingdom tend to identify themselves with the international community rather than with the British L2 speech community. Additionally, it has become increasingly more difficult for the students to identify a clear target group or culture in such a multilingual and multicultural global community. English as a world language connects the learners with foreign countries, and with people with whom they can communicate in English. Through interactions with native as well as non-native English speakers, the learners are immersed in an international relationship as members of the global community, and might generate possible selves speaking with international students, pursuing an international career, or working in a foreign country. From this perspective, international posture is shown to be a powerful motivator in enhancing the students’ WTC and to expand the frequency of communication as well as making more effort in English learning. In other words, from the perspective of emotioncy, it can be said that international posture allows the students to experience a variety of senses (heard, seen, used or created; Pishghadam et al., 2015) when they are engaging relevant English communication and learning activities, and consequently, the involved students can develop proximal emotions which are close to the reality (Pishghadam et al., 2015), not only in terms of different concepts in English language, but also the senses of their self-related images as members of the international community. Moreover, the finding from this study was consistent with Yashima’s (2000) research that instrumental orientation, together with intercultural friendship orientation, significantly predicted motivational intensity to learn English, which in turn can lead to greater language proficiency.

### The Substantial Opposite Effects of Ideal L2 Self and Ought-To L2 Self

Different from the observation by Henry and Cliffordson (2017) about the less powerful role of future self-guidance in aligning a learner’s motivated behavior in highly globalized settings, finding from the current study highlighted the pivotal role of ideal L2 self in such learning context. The study revealed the substantially important role of the ideal L2 self in determining the Chinese overseas students’ motivated learning behaviors, in which the ideal L2 self has significant relations with L2 WTC and frequency of communication, and a relative strong relationship with intended learning effort. However, the ought-to L2 self displayed a substantial negative or insignificant relationship with all three kinds of motivated learning behaviors; in other words, the stronger the ought-to L2 self that a person expresses, the less likely the person will be motivated in L2 learning. This finding is intriguing. On the one hand, in line with an array of prior studies that reported the significant power of the ideal L2 self in predicting and explaining motivated learning behaviors (Magid, 2009; Kim and Kim, 2014; Smith et al., 2020), it is not surprising that intrinsic interest and a strong self-concept embodied in the construct of the ideal self are more powerful in boosting language learning engagement than extrinsic motivations (Deci and Ryan, 1985, 2002).

On the other hand, distinct from the previous studies that proved the vital (Lockwood et al., 2005) or limited role (You and Dörnyei, 2016) of the ought-to L2 self in L2 learning for Chinese students, the current study indicates that the ought-to L2 self was not helpful in promoting the Chinese overseas students’ L2 motived learning behaviors. Probably this could be explained by considering the change of learning context. In accordance with what previous studies identified (Li, 2014), the Chinese students studying in the United Kingdom had increased opportunities to interact with native or non-native speakers of English in their daily lives. In this case, the negotiation of meaning involved in interactions (DeKeyser, 2007) allows the learners to see, hear, and use English in a straightforward way, which is more helpful in shaping a tangible desired image as a competent English user immediately, and in turn may boost willingness to speak in English. In other words, the learners are more likely to move to involvement or metavolvement (Pishghadam et al., 2015) with active motivation that they are directly experiencing or even creating the language when they engaged in the L2 interaction. As result, the emotions and the sensory inputs the individuals received from the communication environment can work as sensory capital (Pishghadam et al., 2019b; Pishghadam and Shakeebaee, 2020) in developing their understanding of the language as well as perception of their ideal future-self-images more directly, which, in turn, may evoke their willingness to participate in English language learning and communication.

Comparatively, the orientation of meeting significant others’ expectations or avoiding negative outcomes seemed less conducive in encouraging one’s WTC, and might even inhibit students’ motives and intended effort in engaging L2 interaction and thereof relevant L2 learning. That is to say, rather than inducing active incentives, ought-to L2 self in this case becomes a demotivating factor that is not helpful in evoking the language learners’ senses and emotions which can facilitate them to move from exvolvement to involvement in the activity they are performing. To some extent, the findings might indicate the importance of lessening language learning pressure perceived by language learners, named “the anti-ought-to self,” observed in previous studies (e.g., Thompson and Vásquez, 2015; Liu and Thompson, 2018), and highlight the meaningfulness of motivating demotivation in language education, especially in the learning context of study abroad (Zeynali et al., 2019; Pishghadam et al., 2021).

### The Mediation of Instrumentality and Parental Encouragement in Learners’ Intended Learning Effort

Although instrumentality cannot be singled out as the principal motivational parameter, data from the study shows its strong correlations with students’ intended learning effort. Considering the language learning context of studying abroad, it is not difficult to understand the tight interrelationship between instrumental orientation and using a second language; studying in the United Kingdom requires more pragmatic use of the English language in meeting personal demands and goals in their daily life, as well as preparing themselves for pursuing a career in the future. The students in this study tended to invest more effort in L2 learning, including acquiring new vocabularies and reading academic articles, in order to being able to communicate more effectively in English or to gain a well-paid and promising job.

It is intriguing that parental encouragement does not show strong links with both L2 WTC and frequency of communication, but it is strongly correlated with intended learning effort. The result may reveal that with the change of the learning environment, the intervention from parents is still significant in guiding the learners’ stated *intentions* to invest effort in learning English but plays a much less prominent role when it comes to the students’ reported engagement in *actual communication in L2*. In other words, it can be said that parental encouragement tends to play a role positively in evoking their children’s active demotivation that deals with performing L2 learning but with less mental engagement or passive motivation that concerns thinking rather than doing; however, it is difficult in shaping the learners’ active motivation that attach with higher emotioncy levels, i.e., involving in actual communication in English. This, to some extent, could reflect that, without the chance to oversee their children directly, parents’ encouragement seems not to have much influence on communication-related L2 actions that normally involve a host of situated factors over which parents no longer have any control, but requires individuals’ higher level of agency to sense them. At the same time, this to a certain extent supports the Chinese imperative as a motivator identified from previous studies (Chen et al., 2005; Taguchi et al., 2009). The required motivation (Warden and Lin, 2000) appears to be no longer effective in some cases of L2 communication, for instance, when assignments that involve frequent communications and group work outside the classroom become one of the main assessments instead of high-stake tests and exams that usually require an individual’s intensive learning efforts in full preparation in the new academic culture. On the other hand, the required motivation with parental encouragement in particular tends to be internalized and retained from students’ past learning experiences, and could still impact the effort that students intend to invest in persistent English language learning. For example, the students intend to invest more effort in gaining vocabularies, reading, and writing in order to complete assignments successfully. In this sense, the current study offered evidence to support the Chinese imperative as a motivator for the Chinese overseas students grounded in the culturally specific Chinese setting. More importantly, the study extends understanding of parental encouragement by showing its persistent influence on the Chinese overseas students’ intended effort regarding English language learning in a study abroad context. However, it is also noteworthy that the role of parents as envolvers in learners’ L2 learning in the study abroad context is less powerful compared to its influence identified in previous studies conducted in the Chinese sociocultural context, to explicate, it seems to be more helpful in developing the language learners’ passive motivation rather than active motivation.

### Implications for Practice and Research

These results offer practical implications in English language education in respect of sustaining language learners’ L2 motivation during study abroad. The crucial role of international posture on learners’ motivated L2 learning suggests that cultivating language learners’ positive attitudes, interest in international culture and affaires, and a nuanced and comprehensive appreciation of the global world surrounding them, should be the primary goal in L2 teaching for students who are planning to study abroad, who are currently experiencing international mobility, and even those who take general education in schools and participate in general examinations but are embedded in the increasingly globalized world. However, teaching content and methods may need to be adjusted according to principles of different institutional settings. In addition, the findings also indicate that teachers as envolvers are expected to carry out various emotional roles (Pishghadam et al., 2019b), and have responsibilities to facilitate students in enhancing their awareness of their learners’ emotions in visualizing their idealized future self-images in relation to global citizens by using different resources and techniques throughout the teaching process. Dörnyei and Kubanyiova (2014) and Jones (2020) proposed a couple stages and the MUSIC model of motivation with an array of techniques in supporting and encouraging students to build realistic and positive visions in the language classroom, e.g., training imagery skills, creating narrative tasks in teaching routine, and encouraging students to keep learning journals. In conjunction with teacher stroke beheviors with positive verbal or non-verbal stroking cues, like smiling or using expression of I love you, these practices value understanding of students’ presence and individual needs, and therefore could be helpful in increasing their emotioncy levels and boosting and their motivation from passive to active. In this way, teachers can adopt an evolving role in preparing the language learners with “Best Possible Self” (Altintasa et al., 2020) that as a high level of sensory capital could maximize their gains for the stay abroad. However, this goal might be difficult to accomplish without clear-cut guidelines at different institutional levels as well as efficient cooperation with student’s parents, so that instructors and coordinators could be more aware of their responsibilities, and parents could be cognizant about aiding their children in fulfilling achievements in a range of personal, social, and academic arenas, thus enhancing the true benefits that language learners can gain from international mobility.

## Conclusion and Limitations

This study draws a holistic picture about Chinese overseas university students in the United Kingdom who are motivated by a complex of variables with varying impact in their English language learning beyond the classroom. Among these motivational variables, international posture is the most dominant and powerful component in determining the students’ motivated learning actions. Additionally, the ideal L2 self also plays a significant role as an incentive in motivating Chinese learners’ participation in L2 communication, which conforms to the critical role of the ideal L2 self that is typically considered as a core component in enhancing motivational actions; at the same time, the ought-to L2 self demonstrated with limited positive influence on Chinese learners in the Chinese sociocultural context has negative impact on influencing the students’ motivation in the current study abroad investigation context. What is more, instrumentality and parental encouragement in the Chinese imperative that is particularly shaped within the Chinese culturally specific context only played important roles in learners’ intended learning effort. The study indicates that parental encouragement is still a powerful construct in incentivizing the Chinese student’s intentional English learning effort in a study abroad context; however, the degree of significance may vary according to individuals and learning situations. Finally, the motivational variable of attitudes to L2 speakers/community and culture that showed a significantly powerful motivating impact on language learners’ L2 learning in previous studies was not prominent in this study. The current study offers additional support for the argument that a diversity of reasons for learning a language should be taken into account in examining L2 motivation (Duff, 2017; Li, 2021).

The findings of this study need to be considered with caution due to its limitations. The small sample size may not be representative of Chinese overseas students in general, thus the findings could not be generalized to other Chinese overseas students in different contexts. Further inquiries with a variety of L2 learner populations in a wide range of language learning settings would be helpful to better understand differences in the motivation of learners of English or other second languages in a study abroad context. Moreover, participants in this study are all university postgraduates, in other words, they share one social role as student; the effect of social role obligation cannot be generalized to other social identities, thus further research considering participants with less academic orientation, e.g., those are already in the workforce, could be useful in shining more light on understanding different L2 motivational profiles of study abroad experiences. Mixed methods research adopted in the current study allows a more comprehensive understanding on language learners’ English language learning motivation in a study abroad context, future L2 study abroad research may benefit from longitudinal designs using multiple data collection methods for more nuanced investigation at different points in time.

## Data Availability Statement

The raw data supporting the conclusions of this article will be made available by the authors, without undue reservation.

## Ethics Statement

The studies involving human participants were reviewed and approved by The Ethics Committee, University of Birmingham (ERN_11–1,150). Written informed consent for participation was not required for this study in accordance with the national legislation and the institutional requirements.

## Author Contributions

ZY: conceptualization, methodology, formal analysis, investigation, resources, data curation, writing (original, review, and editing), and visualization. KZ: methodology, investigation, resources, data curation, writing (review and editing), visualization, and supervision. YM and XQ: writing (review and editing) and supervision. LW: writing (review and editing). All authors contributed to the article and approved the submitted version.

## Funding

Authors thank the financial aids from Chinese Postdoctoral Science Foundation (Grant No. 2017M623140); Special Foundation of Economic Research Center of Buchang Xixian (Grant No. SMZX202111).

## Conflict of Interest

The authors declare that the research was conducted in the absence of any commercial or financial relationships that could be construed as a potential conflict of interest.

## Publisher’s Note

All claims expressed in this article are solely those of the authors and do not necessarily represent those of their affiliated organizations, or those of the publisher, the editors and the reviewers. Any product that may be evaluated in this article, or claim that may be made by its manufacturer, is not guaranteed or endorsed by the publisher.
